# Supplementary material for: Potential Global Distribution of Daktulosphaira vitifoliae under Climate Change Based on MaxEnt
Source: Insects. 2021 Apr 13;12(4):347. doi: 10.3390/insects12040347 (PMC8069807; doi:10.3390/insects12040347)
Supplement: Supplementary file 1 [file insects-12-00347-s001.zip › insects-1083998-fsup/Supplementary/Table S1.docx]

**Table S1**

**References**

Hoffmann M, Ruehl EH, Eisenbeis G, Huber L. 2015. Indications for rootstock related ecological preferences of grape phylloxera. Vitis, 54: 137-142.

Kim CS, Lee CH, Park HS, Lee GP. 2005. In vitro grafting of grape with phylloxera resistant rootstock cultivars. Vitis, 44(4): 195-196.

Madalon FZ, Lima VLS, Pratissoli D, Meneghelli CM, Carvalho JR, Zucoloto M. 2018. Occurrence of grape phylloxera *Daktulosphaira vitifoliae* Fitch (Hemiptera: Phylloxeridae) in the state of Espirito Santo, Brazil. Journal of experimental agriculture international, 20(3): 1-5.

Al-Antary TM, Nazer IK, Qudeimat EA. 2008. Population trends of Grape Phylloxera, *Daktulospharia (Vites) vitifoliae* Fitch. (Homoptera: Phylloxeridae) and effect of two insecticides on its different stages in Jordan. Jordan Journal of agricultural science, 4(4): 343-349.

Islam MS, Roush TL, Walker MA, Granett J, Lin H. 2013. Reproductive mode and fine-scale population genetic structure of grape phylloxera (*Daktulosphaira vitifoliae*) in a viticultural area in California. BMC Genetics, 14(123): 1-11.

Corrie AM, Crozier RH, Heeswijck RV, Hoffmann AA. 2002. Clonal reproduction and population genetic structure of grape phylloxera, *Daktulosphaira vitifoliae*, in Australia. Heredity, 88: 203-211.

Corrie AM, Hoffmann AA.2004. Fine-scale genetic structure of grape phylloxera from the roots and leaves of Vitis. Heredity, 92: 118-127.

Downie DA. 2005. Evidence for multiple origins of grape phylloxera (*Daktulosphaira vitifoliae* Fitch) (Hemiptera: Phylloxeridae) in South African vineyards. African Entomology, 13(2): 359-365.

Corrie AM, Robyn VH, Hoffmann AA. 2003. Evidence for host-associated clones of grape phylloxera *Daktulosphaira vitifoliae* (Hemiptera: Phylloxeridae) in Australia. Bulletin of Entomological research, 93(3): 193-201.

Forneck A, Mammerler R, Tello J, Breuer M, Muller J, Fahrentrapp J. 2019. First European leaf-feeding grape phylloxera (*Daktulosphaira vitifoliae* Fitch) survey in Swiss and German commercial vineyards. European Journal of Plant Pathology, 154: 1029-1039.

Forneck A, Walker MA, Blaich R. 2000. Genetic structure of an introduced pest, grape phylloxera (Daktulosphaira vitifoliae Fitch), in Europe. Genome, 43: 669-678.

Bao LV, Scatoni IB, Gaggero C, Gutierrez L. Monza J, Walker MA. 2015. Genetic diversity of grape phylloxera leaf galling populations on vitis species in Uruguay. American Journal of Enology and Viticulture, 66(1): 46-53.

Tello J, Forneck A. 2019. Use of DNA Markers for Grape Phylloxera population and evolutionary genetics: from RAPDs to SSRs and Beyond. Insects, 10(317): 1-21

Riza S, Lund K, Lin H, Walker MA. 2014. Development and characterization of a large set of microsatellite markers for grape phylloxera (*Daktulosphaira vitifoliae* Fitch). Vitis, 53(2): 95-101.

Tello J, Mammerler R, Cajic M, Forneck A. 2019. Major outbreaks in the nineteenth century shaped grape phylloxera contemporary genetic structure in Europe. Scientific reports, 9: 17540.

Vidart MV, Mujica MV, Bao L, Duarte F, Bentancourt CM, Franco J, Scatoni IB. 2013. Life history and assessment of grapevine phylloxera leaf galling incidence on Vitis species in Uruguay. SpringPlus, 2: 181.

Zhao JJ, Gao D, Feng JN. 2015. Potential geographic distribution in *Daktulosphaira vitifoliae* Fitch in China based on MaxEnt model. Journal of northwest A & F University, 43(11): 99-112.

Mao F, Yao HM, He YY, Chen K, Yu H, Liu ZH, Qin H. 2014. The potencial geographical distribution of *Daktulosphaira vitifoliae* based on CLIMEX in China. Acta environmental entomology, 36(3): 293-297.
