# Supplementary figures and images for: Potential Global Distribution of Daktulosphaira vitifoliae under Climate Change Based on MaxEnt
Source: Insects. 2021 Apr 13;12(4):347. doi: 10.3390/insects12040347 (PMC8069807; doi:10.3390/insects12040347)

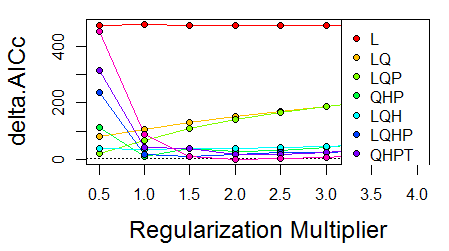

Supplement: Supplementary file 1 [file insects-12-00347-s001.zip › insects-1083998-fsup/Supplementary/Figure S1.tiff]

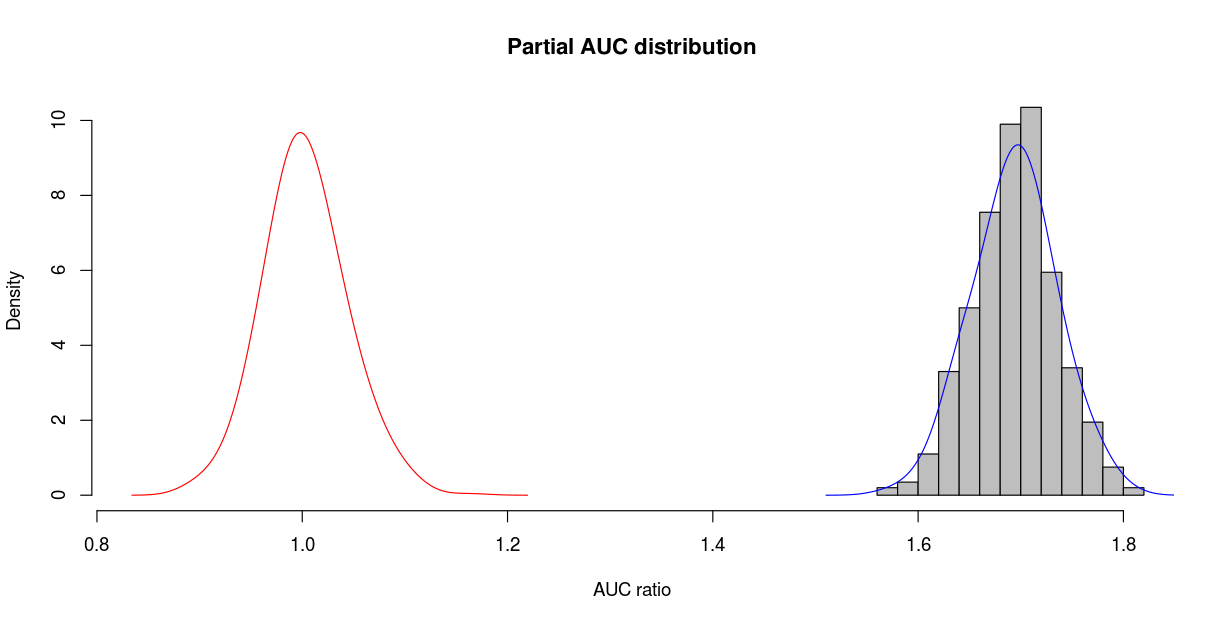

Supplement: Supplementary file 1 [file insects-12-00347-s001.zip › insects-1083998-fsup/Supplementary/Figure S2.png]
